# X-ray scintillator lens-coupled with CMOS camera for pre-clinical cardiac vascular imaging—A feasibility study

**DOI:** 10.1371/journal.pone.0262913

**Published:** 2022-02-11

**Authors:** Swathi Lakshmi Balasubramanian, Ganapathy Krishnamurthi

**Affiliations:** Department of Engineering Design, Indian Institute of Technology-Madras, Chennai, TamilNadu, India; Rutgers University Newark, UNITED STATES

## Abstract

We present the design and characterization of an X-ray imaging system consisting of an off-the-shelf CMOS sensor optically coupled to a CsI scintillator. The camera can perform both high-resolution and functional cardiac imaging. High-resolution 3D imaging requires microfocus X-ray tubes and expensive detectors, while pre-clinical functional cardiac imaging requires high flux pulsed (clinical) X-ray tubes and high-end cameras. Our work describes an X-ray camera, namely an “optically coupled X-ray(OCX) detector,” used for both the aforementioned applications with no change in the specifications. We constructed the imaging detector with two different CMOS optical imaging cameras called CMOS sensors, 1.A monochrome CMOS sensor coupled with an f1.4 lens and 2.an RGB CMOS sensor coupled with an f0.95 prime lens. The imaging system consisted of our X-ray camera, micro-focus X-ray source (50kVp and 1mA), and a rotary stage controlled from a personal computer (PC) and LabVIEW interface. The detective quantum efficiency (DQE) of the imaging system(monochrome) estimated using a cascaded linear model was 17% at 10 lp/mm. The system modulation transfer function (MTF) and the noise power spectrum (NPS) were inputs to the DQE estimation. Because of the RGB camera’s low quantum efficiency (QE), the OCX detector DQE was 19% at 5 lp/mm. The contrast to noise ratio (CNR) at different frame rates was studied using the capillary tubes filled with various dilutions of iodinated contrast agents. In-vivo cardiac angiography demonstrated that blood vessels of the order of 100 microns or above were visible at 40 frames per second despite the low X-ray flux. For high-resolution 3D imaging, the system was characterized by imaging a cylindrical micro-CT contrast phantom and comparing it against images from a commercial scanner.

## Introduction

X-ray imaging systems have broad applicability in preclinical imaging studies. These range from cardiac angiography in mice using fast frame rate X-ray cameras and pulsed X-ray tubes to high-resolution anatomical imaging with micro-focus X-ray tubes and flat-panel detectors [1–6]. Typically, angiographic studies involve the sequential scanning of the heart after injecting an iodinated contrast agent (ICA) into the rodent’s tail vein. A combination of high-resolution micro-CT and angiography studies helps in obtaining both structural and functional information.

Digital subtraction angiography (DSA) study on mice with a clinical X-ray tube gives excellent visualization of cardiac arteries [7]. In addition, one such study also reported the usage of a high concentration of ICA (≈ 623mg-I/ml) [8]. In many high-end systems, i.e., systems offering high resolution, X-ray cameras consist of an X-ray scintillator either optically coupled or directly coupled with optical fibers to a cooled CCD camera [19]. Most systems offering high frame rates for angiography study utilize pulsed clinical X-ray tubes with large focal spots. It limits the resolution of the system unless coupled with a high-resolution flat panel detector(FPD) with the rodent placed right over the detector or camera to overcome focal spot blurring. X-ray imaging systems with micro-focus tubes or nano-focus tubes for high-resolution 3D imaging cannot offer the flux to perform fast frame rate imaging required for cardiac angiography like studies in small animals (heart rates > 100 beats per minute). Also, many cooled CCD cameras can do fast frame rate imaging only with pixel binning.

To summarise, the imaging system requirements for fast frame rate and high-resolution imaging are vastly different, leading to different setups. The use of clinical X-ray tubes also leads to increased radiation safety infrastructure requirements. Similarly, high-resolution X-ray cameras also increase the overall cost of the system but limit the applications.

We have tabulated a list of X-ray imaging studies using scintillators coupled to low light imaging cameras in Table 1. Our work is unique, using a simplified and inexpensive setup for both angiography and high-resolution rodent imaging. In this work, we leverage the fast frame rates allowed by off-the-shelf CMOS cameras (RGB and grayscale) to perform angiography studies using a micro-focus X-ray tube as a source in addition to high-resolution 3D imaging. We study the feasibility of using such a setup for performing both angiography-like studies and high-resolution 3D imaging. We characterize the system in terms of detective quantum efficiency (DQE) using a cascaded linear model and contrast to noise ratio (CNR) using phantom studies. We perform both phantom and in-vivo studies to assess and suggest measures to improve the system’s performance. Our work presented one of a kind study to leverage off-the-shelf components to perform both rodent angiography studies and high-resolution 3D imaging, without the need for specialized X-ray sources and cooled CCD cameras in addition to being inexpensive.

**Table 1:** Overview of design and characterisation of CCD/CMOS based X-ray detector.

Paper	Imaging system	Proof of concept
Helen XF, (2015) [9]	• Phosphor screen of size 24x36 mm	Direct coupling: experiments with models
	• DSLR camera, Andor neo, PIXIS 2048B	
	• f1.4 lens, MTF 50% at 2.5 lp/mm, 30 fps	
	• Operating energy: 130 kVp, 0.5 mA	
Yong R et al., (2014) [10]	• Carbon fiber substrate CsI of size 431x431 mm	Indirect coupling: chest X-ray imaging
	• Mirror and lens coupling with CCD camera	
	• Spatial resolution (planar imaging) 4.5 lp/mm	
Koch A (1994) [11]	• Screen *Gd*_2_ *O*_2_ *S*:Tb	Indirect coupling: characterisation of detector
	• Mirror and lens coupled with CCD camera	
	• f0.87 lens, MTF 20% at 5 lp/mm	
	• Operating energy 10–20 keV	
Jakob CL et al., (2016) [12]	• Scintillator fiber coupled with CCD camera	Characterisation of various scintillators (Princeton inst.)
	• No optical coupling, Gadox, CsI:Tl(400 *μ*m)	
	• MTF 10% and DQE < 0.1 at 10 lp/mm, 38 keV	
Uesugi K et al., (2011) [13]	• Gadox scintillator, f1.65 and f2.4	Lens and fiber coupled detector comparison study
	• Lens coupling efficiency ≈ 0.0076 @ 21 keV	
	• pixel size (lens coupled) 17.1 *μ*m,	
Yang M et al., (2006) [14]	• Gadox coupled with image intensifier and CCD	Indirect coupling: in-vivo animal (CT) study
	• Intrinsic pixel size 12 *μ*m, 18 fps	
	• Imaging characteristics compared with FPD	
Xie H et al., (2019) [15]	• YAG:Ce and LuAG:Ce scintillator	Indirect coupling: synchroton source imaging
	• Optical coupling efficiency 0.15	
	• spatial resolution 10 *μ*m	
	• 80000 fps, operating energy: 15–35 keV	
Jain A et al., (2011) [16]	• CsI:Tl(300 *μ*m) coupled with two stage image intensifiers, taper fiber(2.88:1) and CCD	Direct coupling: characterisation image of stent in rabbit
	• MTF ≈ 0.08 & DQE < 0.1 @ 5 lp/mm	
	• Quantum detection efficiency of CsI:Tl ≈ 0.62	
	• 30 fps, X-ray source 54 kVp	
Srinivasan V et al., (2004) [17]	• CsI:Tl(1:1) fiber coupled with interlined CCD	Direct coupling: study with various thickness of scintillator
	• For CsI:Tl(450 *μ*m), 78 *μ*m pixel pitch	
	• MTF ≈ 0.1 and DQE < 0.1 @ 5 lp/mm	
	• 30 fps, X-ray source 72 kVp	
Ganguly A et al., (2003) [18]	• CsI:Tl(250 *μ*m) fiber coupled(1.8:1) with CCD	Direct coupling: characterisation of detector
	• Quantum detection efficiency of CsI:Tl ≈ 0.61	
	• MTF ≈ 0.1 & DQE < 0.12 @ 5 lp/mm	
	• 5 fps, X-ray source 80 kVp	

## Materials and methods

### Design of the OCX detector

**Fig 1:**
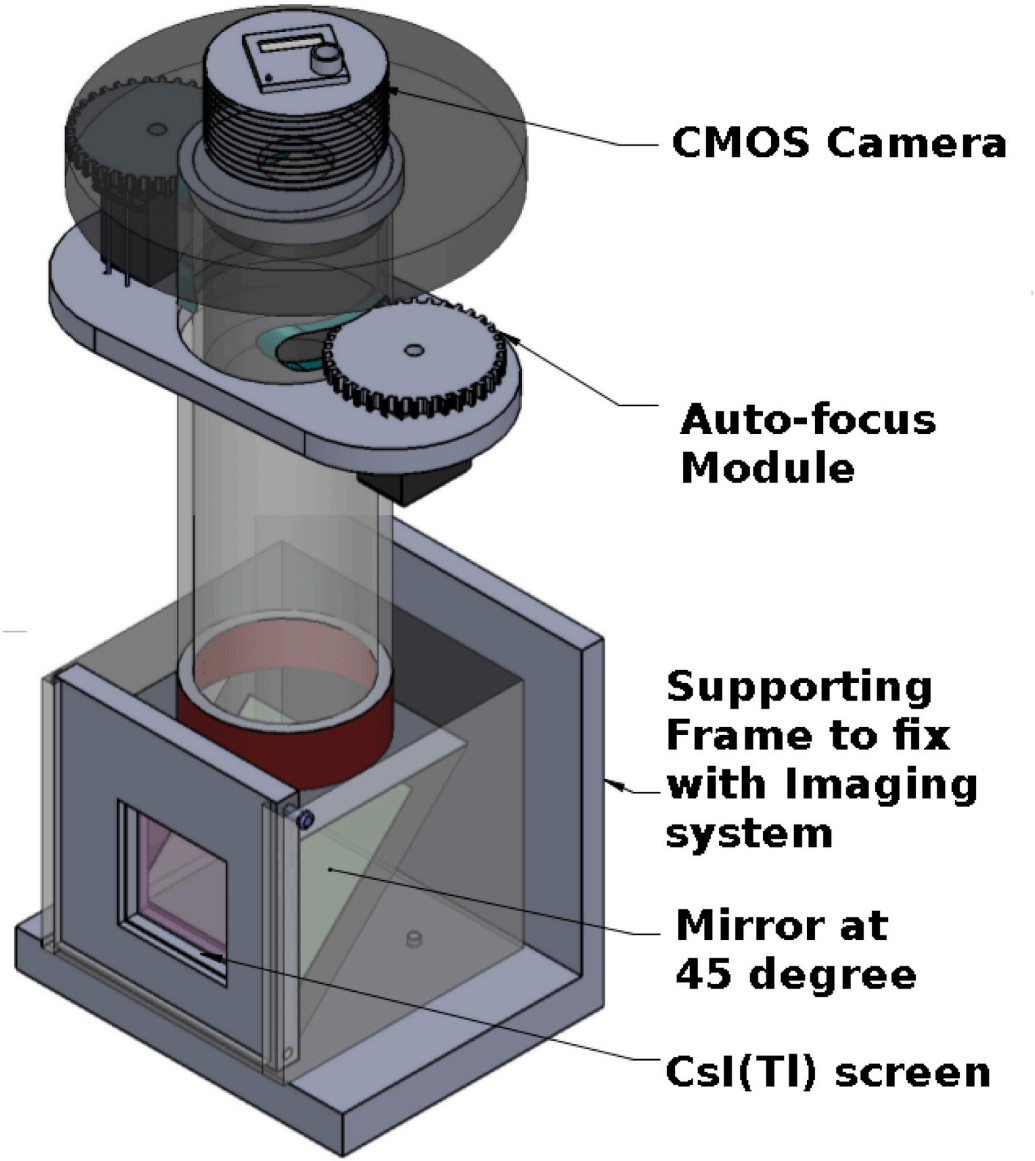
Optically coupled X-ray detector. 3D view of OCX detector designed with an inexpensive monochrome/RGB CMOS sensor. The CsI(Tl) scintillator screen converts the incident X-rays to light photons. The light photons were reflected by the mirror and collected by the lens coupled with the CMOS sensor. The auto-focus module adjusts the aperture and focus of the lens through the LabVIEW user interface.

The X-ray camera consists of a CsI(Tl) scintillator of 50x50 mm (J13113, Hamamatsu, Japan) to receive the exit X-ray flux from the sample. Between the scintillator and CMOS camera, an aluminized mirror (75x75 mm) was positioned at an angle of 45° (Fig 1). The system allows to couple any CMOS sensor. Experiments were performed with two different CMOS sensors (Table 2). One was a monochrome camera (BFLY-U3–23S6M-C Point Grey inc., USA) coupled with an f1.4 lens (Nikor 50mm prime lens, Japan), and the other was an RGB camera (GS3-U3–23S6C-C Point Grey inc., USA) interfaced with an f0.95 lens (Schneider 25mm prime lens, Edmund optics). The focus and aperture of the lens were coupled to the servo motors using rubber-made teeth belts, and it’s shown as an auto-focus module in Fig 1. The lens parameters (focus and apeture) can be adjusted using the gear mechanism and triggered through the LabVIEW interface software.

**Table 2:** Point grey camera (CMOS sensor) physical parameters.

	*BFLY-U3–23S6M-C*	*GS3-U3–23S6C-C*
Pixel size	5.86 x 5.86 *μ*m	5.86 x 5.86 *μ*m
Active area	1920 x 1200 pixels	1920 x 1200 pixels
Quantum efficiency	82% at 525 nm	59.7% at 530 nm
A/D conversion	10 bits per pixel	10 bits per pixel
Exposure time	0.019 ms to 3.9 s	0.005 ms to 31.9 s
Read out noise	13.77 electrons	6.9 electrons
Pixel capacity	33158 electrons	32291 electrons
Frame rate	1–41 fps	1–163 fps
Sensor area focused by OCX detector	6.3 x 6.3 mm	6.0 x 6.0 mm
Spatial resolution of OCX detector	44.64 *μ*m	53 *μ*m
Field of view CsI(Tl) screen	47.6 mm	40.7 mm

We tabulated the specifications of the monochrome and RGB camera in Table 2. Also, we included the theoretically obtained parameters after integrating the camera into the OCX detector [9].

For the f1.4 lens, the numerical aperture (NA) was 0.357, diameter of the entrance pupil (D_*ep*_) was 35.7 mm and lens collection efficiency (*η*_*lens*_) was 9.96%. Similarly for f0.95 lens, NA = 0.53, D_*ep*_ = 26.3 mm and *η*_*lens*_ = 18.8% [9].

### Characterisation of the OCX detector

#### Cascaded linear model of the OCX detector

**Fig 2:**
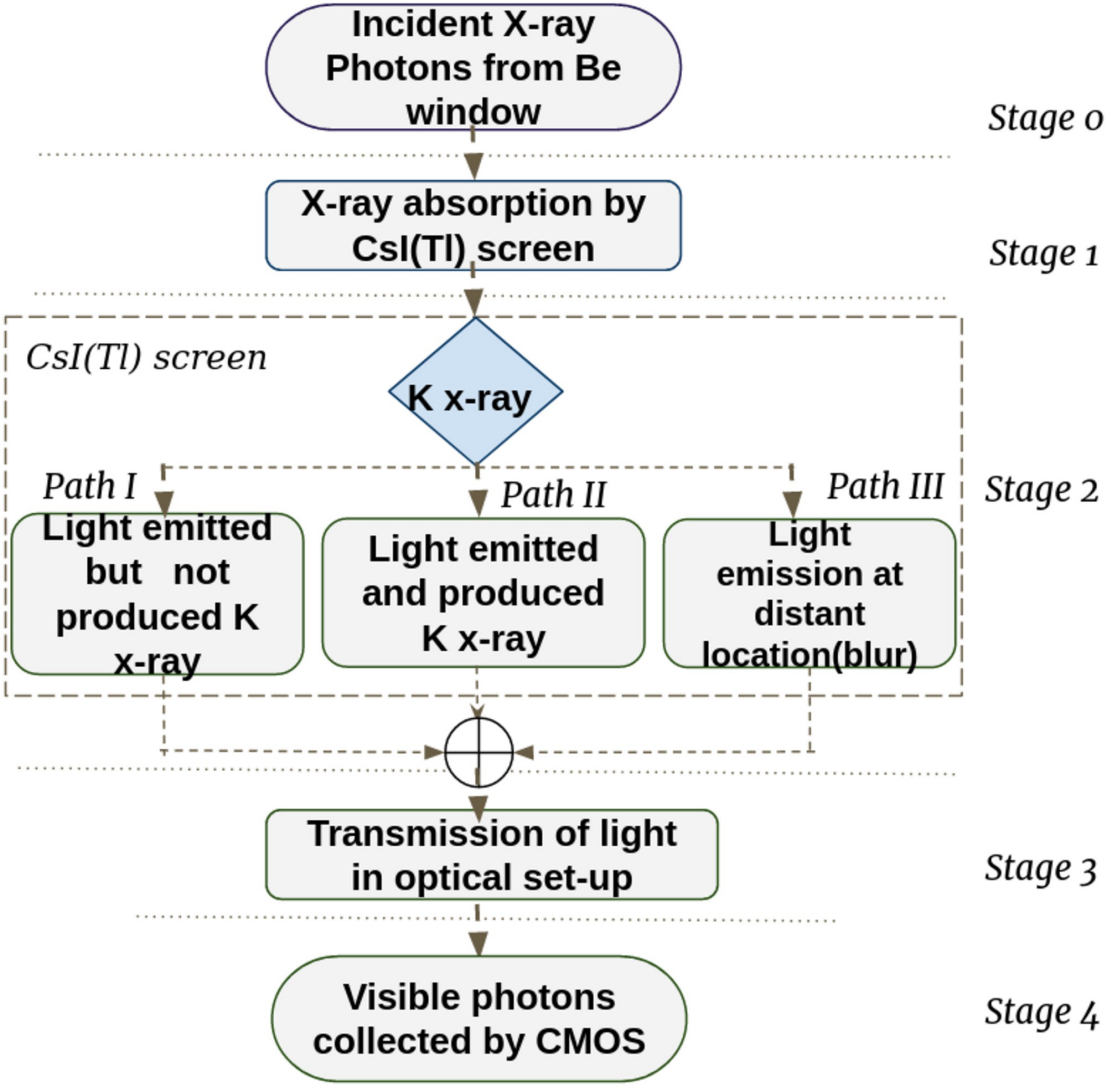
Cascaded linear model of the OCX detector. The cascaded linear model had different stages, from the X-ray incident on the screen until the CMOS sensor received it. We characterized each stage by an absorption/gain factor or MTF characterizing the resulting blur due to the stage.

The light collection efficiency of the OCX detector was estimated using the cascaded linear model (Fig 2) [20]. In *stage 0*, the generated photons from the X-ray tube exits the Be window and these photons interact with the CsI(Tl) screen in *stage 1*. The absorbed photons from the above stage converted to visible light in the *stage 2* [21]. These visible photons get attenuated by the optical arrangement in *stage 3* and reach the CMOS sensor unit in *stage 4*.

**Fig 3:**
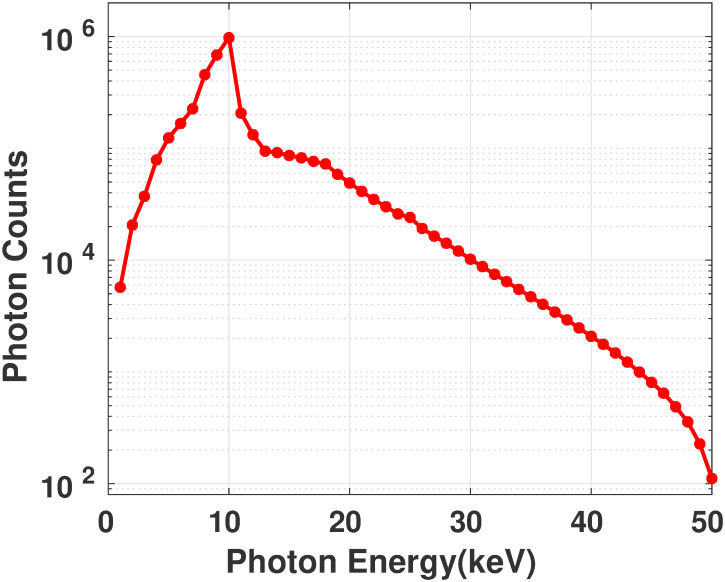
X-ray source spectrum. X-ray spectrum of the micro-focus tube(50 kVp) used in the experiment.

*Stage 0: X-rays from source*. The micro-focus X-ray source had 50 microns focal spot, a beryllium filter of 200 microns, and a tungsten target (SB-50–1k-BW, source-ray inc, USA). The source had a maximum operating voltage of 50 kVp, 1000 *μ*A, and a cone angle of 25 degrees. The X-ray source spectrum provided by the manufacturer is shown in Fig 3.

*Stage 1: X-ray absorption by the CsI(Tl) screen*. The quantum efficiency(*η*) of the CsI(Tl) screen was obtained using Eq 1.

$$\begin{array}{c} \eta =1-{\text{exp}}^{-\mu (E)x} \end{array}$$ (1)

where *x* was the thickness of the scintillator and *μ*(*E*) was the linear attenuation coefficient at energy E. The efficiency of the CsI(Tl) screen was estimated using 75% of its material density value (4.51 g/cm^3^). The average quantum detection efficiency of the CsI(Tl) screen was given by multiplying the incident number of photons(q_0_) on the screen and the quantum efficiency(*η*) [16].

*Stage 2: X-rays to light conversion*. X-rays incident on the screen undergoes photoelectric interactions such as absorption of X-rays without emission of characteristic K X-rays(I), with emission of characteristic K X-rays(II), and the K X-rays reabsorbed at a distant location(III). Paths I, II, and III represent the interaction happening inside the CsI(Tl) screen [22]. The contrast transfer function (CTF) plotted for various scintillator thicknesses was used to estimate the screen’s modulation transfer function (MTF). The MTF was evaluated by fitting the CTF graph for an unfiltered X-Ray beam (courtesy Hamamatsu) to an exponential equation. Matlab’s (The Mathworks inc. R2019a) curve fitting toolbox was used [18]. The fluorescence probability values from Jain A et al., were used for DQE, signal, and noise power spectrum (NPS) calculations [16]. DQE of the screen shown in Eq 2 was the function of the MTF, noise power spectrum(NPS), and incident photons(q_0_).

$$\begin{array}{c} \begin{array}{c} DQE\left(f\right)=MTF^{2}/\left(q_{0}NPS\right) \end{array} \end{array}$$ (2)

*Stage 3: Optical coupling efficiency*. The light collection efficiency (g_*oc*_) of the optically coupled lens contributes to the output signal to noise ratio (SNR) of the detector. CsI and NaI-based scintillators were highly temperature-dependent [23, 24]. Assuming an extended Lambertian source for the radiographic imaging, the g_*oc*_ of the coupled lens system was given in Eq 3.

$$\begin{array}{c} g_{oc}=T/\left[4\left(F_{no}^{2}\right){\left(1+m\right)}^{2}+1\right] \end{array}$$ (3)

Here *T*, *m* and *F*_*no*_ represents the transmission factor (70–80% for the multi-piece photographic lens), demagnification ratio and F-number of the lens [25]. The blur in the relay lens was not studied separately.

*Stage 4: Absorption of visible photons by the CMOS sensor*. Optical photons(*N*) generated per X-ray photon incident on the CsI(Tl) screen was given by,

$$\begin{array}{c} N=E_{s}(\eta_{screen}/E_{screen}\;in\;eV) \end{array}$$ (4)

where, *E*_*s*_ was the X-ray source energy = 50 keV

*η*_*screen*_ was the maximum energy conversion efficiency of screen = 0.1 [26]

*E*_*screen*_ was the energy of the optical photon at 560 nm.

Substituting the aforementioned values in Eq 4, the number of optical photons(N) per X-ray photon is estimated as 2200 [13]. At 50 kVp, 2200 photons are created by each X-ray photon, and out of which is 32 optical photons reach the CMOS sensor due to g_*oc*_. The light collection efficiency (g_*oc*_) = 0.0145 based on Eq 3. Each optical photon is expected to create 24(19) electrons corresponding to monochrome(RGB) CMOS sensor [15].

#### PSF, NPS and MTF measurement

**Fig 4:**
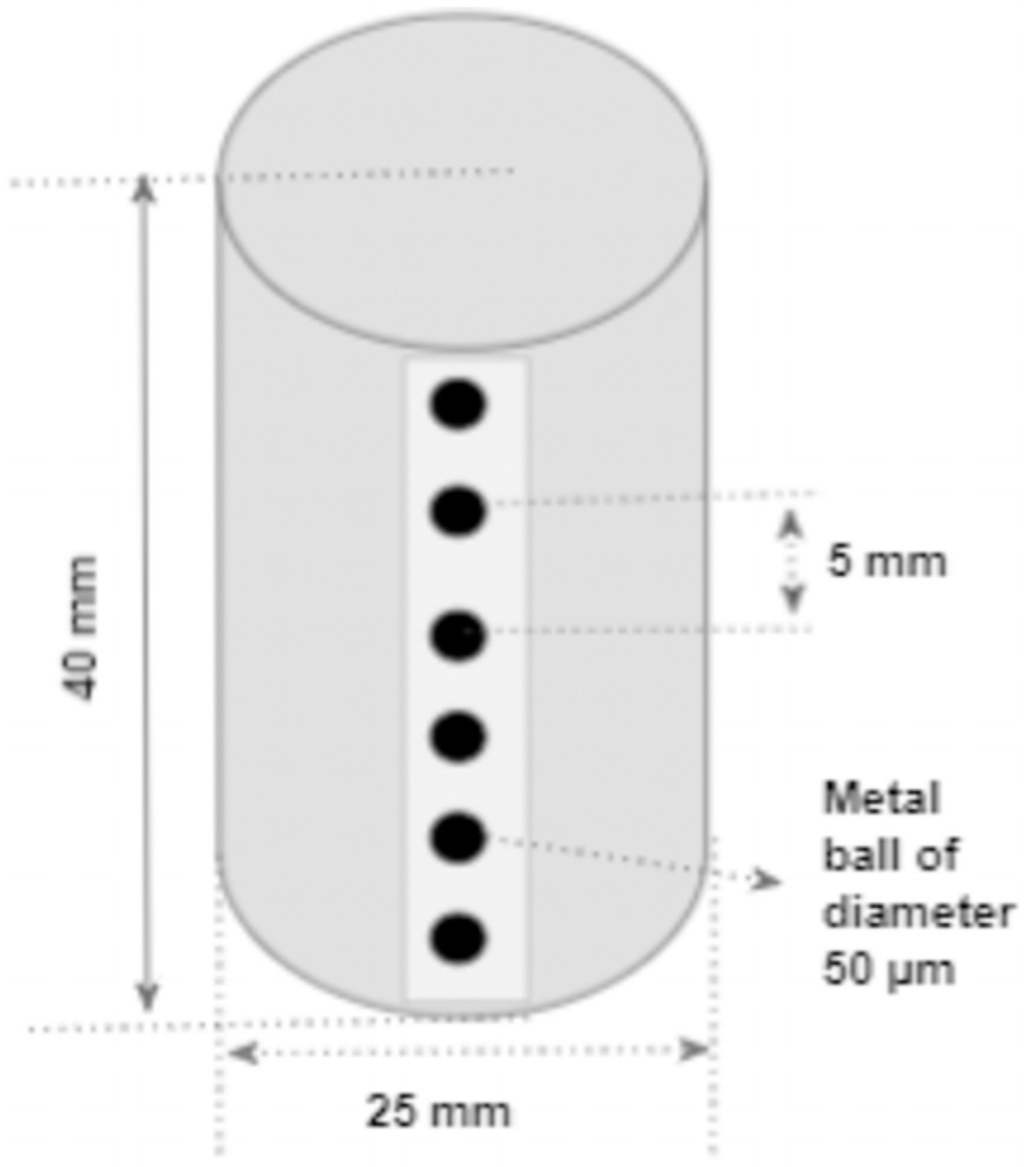
Metal ball phantom. The metal ball phantom is used to perform accurate geometric calibration. The phantom had a series of metal balls of diameter 50 *μ*m taped on the surface of the solid PMMA cylinder (diameter 25 mm).

The detector’s point spread function (PSF) was estimated using the metal ball phantom. The phantom had a steel ball bearing of size 50 *μ*m that was attached to the surface of an acrylic cylinder using tape. A series of metal balls in a single column with the center to center distance of 5mm were taped to the sides of a solid polymethyl methacrylate (PMMA) cylinder (25mm diameter) as shown in Fig 4. The projection image of the aforementioned metal ball phantom was used to analyze the MTF of the imaging detector.

*MTF of the source*. The geometrical unsharpness of the monochrome and RGB OCX detector was obtained using the magnification (*m*) and focal spot size of the X-ray source (50 microns). Here SOD and SDD indicate the distance between source to object and source to detector.

The geometrical unsharpness of the detector due to the source was used to estimate the linear spread function (LSF), and the Fourier transform on the LSF gives the MTF of the source [27]. The LSF of the source was estimated as mentioned in Eq 5.

$$\begin{array}{c} \begin{array}{ccc} LSF(x) & = & 1/\left[\left(m-1\right)*f_{size}\right]\;for\;\left|x\right|\leq 1/2\left(m-1\right)*f_{size}\\ & = & 0;\;otherwise \end{array} \end{array}$$ (5)

*MTF of detector*. MTF of the detector was calculated using the radiographic image of the metal ball phantom(Fig 4). A 32x32 pixel region of interest was selected on the projection image to obtain the point spread function (PSF), and the MTF was estimated from the PSF. The NPS was calculated from the projection images acquired without the object in the field of view. The DQE of the detector was evaluated as a function of MTF and NPS(Eq 2).

#### Spatial resolution measurement

The spatial bar pattern phantom (*QRM* micro-CT phantom, GmbH, Germany) was used to obtain the direct visible measurement of the detector’s spatial resolution. The bar pattern was placed as close as possible to the X-ray tube’s exit window for improved geometric magnification.

### Fast frame rate imaging

**Fig 5:**
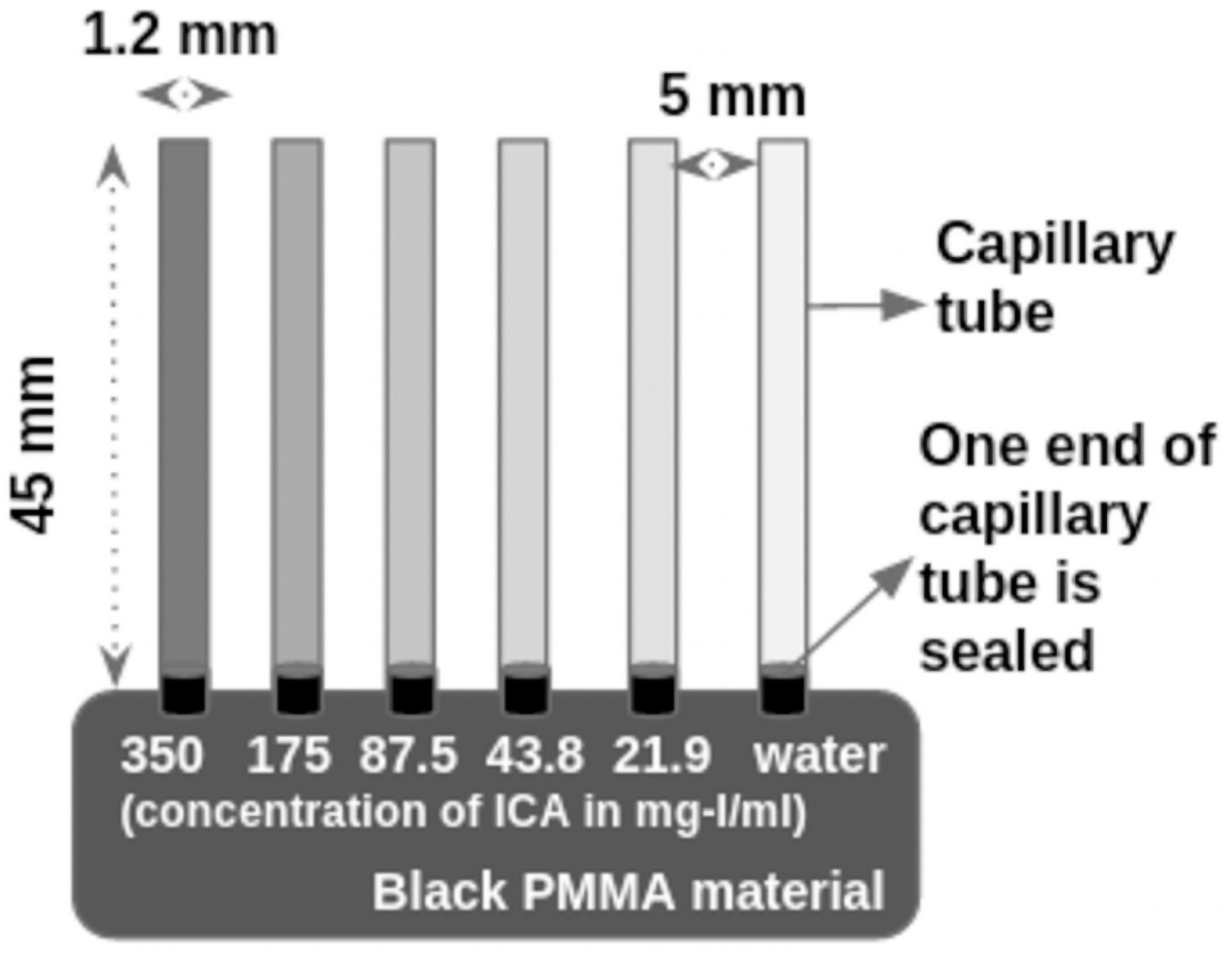
Iodine test phantom to estimate the CNR of the system. The capillary tube of diameter 1.2 mm was filled with different dilutions of ICA (Omnipaque 350 mg-I/ml)-350, 175, 87.5, 43.8, 21.87 mg-I/ml. One end of the capillary was sealed and fixed in the PMMA based material.

Experiments with the iodine test phantom helped to evaluate the detector at high frame rates. The iodine test phantom consists of six capillary tubes (outer diameter 1.2 mm and inner diameter ≈ 1 mm) filled with the different concentrations of iodinated contrast agent (ICA). Capillary tubes were filled with ICA (omnipaque 350 mg-I/ml) concentrations of 350, 175, 87.5, 43.8, 21.87 mg-I/ml, and distilled water. These tubes were sealed at the ends before inserting into an acrylic disc (Fig 5). The capillary tube’s diameter can be considered the same order of magnitude of the mouse aorta, close to the heart [28]. The study estimated the CNR of the system at different exposure times and ICA concentrations.

Apart from the iodine test phantom, the high contrast resolution of the system was estimated using a tungsten wire of 125-micron thickness at different system magnification. A thin W wire of length 50 mm was kept at the center of a solid poly(methyl methacrylate) tube diameter of 30 mm. The thickness of the tungsten wire corresponds to a typical coronary artery in a mouse. So, the tungsten phantom study verifies the visibility of sub-mm structures at high frame rates.

#### Iodine test phantom to estimate the CNR of the detector

Generally, imaging performed at very short exposure of time undergoes errors due to low photon count and electronic noise. The sequence of images was taken at various frame rates (1,10,20,..,87) with an interval of 10. The object visibility of the detector when operated at the high frame rate and constant X-ray energy can be analyzed using two ways:(1) selecting a single image from each frame rate (2D spatial data) and (2) evaluating the set of images with respect to time (3D spatio-temporal data set) [29]. Following the first method, the CNR was evaluated using pixel statistics drawn from hand-marked regions of interest inside the capillary tubes and the background region. In Eq 6, the CNR was estimated as the difference between the mean of the iodinated contrast agent ROI and background intensity ROI divided by the standard deviation of the background intensity ROI. The study acquires projection images at different frame rates with tube voltage set at 50 kVp and tube current at 900 *μ*A. At lower frame rates, specifically at 1fps, the tube current was reduced to 100 *μ*A to avoid saturation. The CNR was evaluated using the following expression in Eq 6.

$$\begin{array}{c} CNR_{r}=\sum_{r=1}^{n}\left(|\mu_{signal}-\mu_{water}|\right)/\sigma_{water}\;\forall \;f \end{array}$$ (6)

Here *μ*_*signal*_ refers to the mean pixel intensity inside capillary tubes filled with iodine contrast, while *μ*_*water*_ refers to the intensity of capillary tube with water. For CNR calculation, multiple small regions of interest *(r = 1,..n)* were drawn inside the capillary tubes, and the mean of the regions was estimated. The experiments were performed at various frame rates(*f*) ranging from 0–90 fps. Beyond 50 fps, the images were noisy, and the capillary tubes could not be visualized.

#### In-vivo small animal coronary studies

*Ethics statement*. All animal experiments were carried out according to the guidelines specified by the Committee for the purpose of control and supervision of experiments on animals, New Delhi (**466/CPCSEA**), and approved by institutional ethics committee approval. Swiss albino mice housed in the animal house facility were brought to our imaging laboratory for performing angiographic studies. Before performing the angiographic study, the animals were anesthetized with a cocktail of ketamine and xylazine. After completing imaging experiments, the animals were allowed to recover and taken back to the animal housing facility. The animals were not sacrificed at the end of the study.

Three swiss albino mice weighing between 20 and 25 grams were used for the angiographic study. A mixture of ketamine (50 mg/ml) and xylazine (23 mg/ml) was used as anesthetizing agent. A custom-built restrainer was used to immobilize the anesthetized mice. Then the sedative mice injected with a tail vein needle were kept safely on the rotary table in the micro-CT system. Approximately 200 *μ*l of iodine contrast agent (Isovue-350 mg-I/ml) was injected into the rodent’s tail vein through a 27-gauge butterfly needle. The X-ray tube was switched *ON*, and 2D projection images were acquired continuously before the ICA injection, and image acquisition continued till the ICA washed out of the rodent’s heart [30].

The diaphragm movement was used to estimate the rodent’s heart, and breath rate as described in Kuntz J et al., [31]. The above method gives approximate breath and beats rate values for animal experiment I. The heart and breath rates of the animals in experiments II and III were estimated from the ECG monitor device [5]. The small animal ECG monitoring device was a simplified setup wherein the leads were fed to the instrumentation amplifier circuit and interfaced with an arduino controller. MATLAB signal processing software denoises and processes the stored ECG data [32].

### Micro-CT imaging

**Fig 6:**
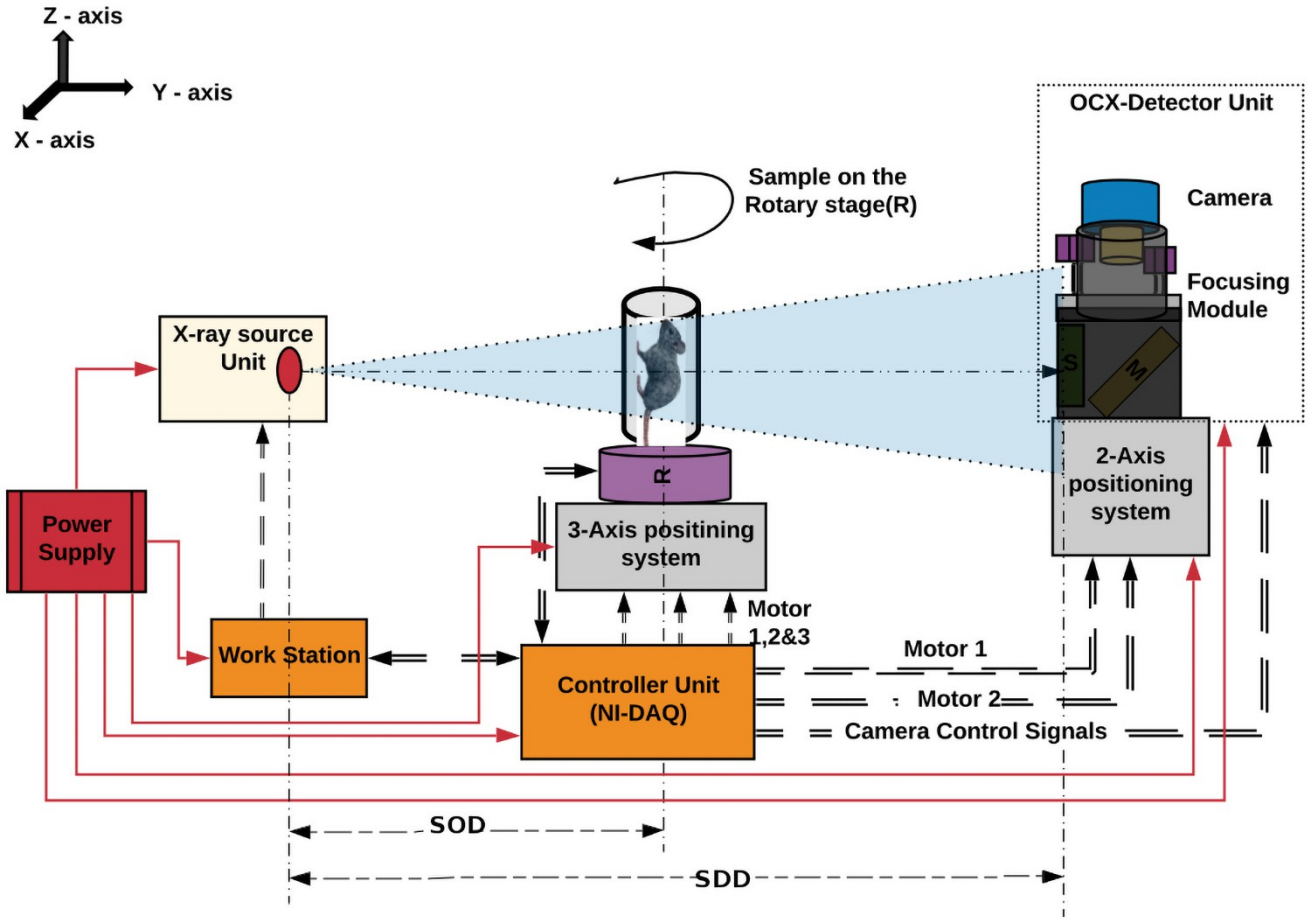
Micro-CT imaging system. Micro-CT imaging system integrated with the OCX detector. Here, SOD and SDD gives the distance between source to object and the distance between source to the detector.

The micro-CT system was comprised of the OCX detector on a two-axis position system, integrated with the microfocus X-ray source (50 kVp, 1000 μA) and a rotary stage for holding the sample and rotating the sample(Fig 6). The sample was rotated in 360° with the help of the rotary stage for tomography studies. The rotary stage was attached to a 3-axis linear positioning system with a travel length of 400 mm along the XY-axis and 100 mm along the z-direction. The LabVIEW interface (NI-DAQ 6341) allows the user to translate the sample stage, set the number of projections for every degree or sub-degree, file location to save the projections data, interface and trigger the camera.

**Fig 7:**
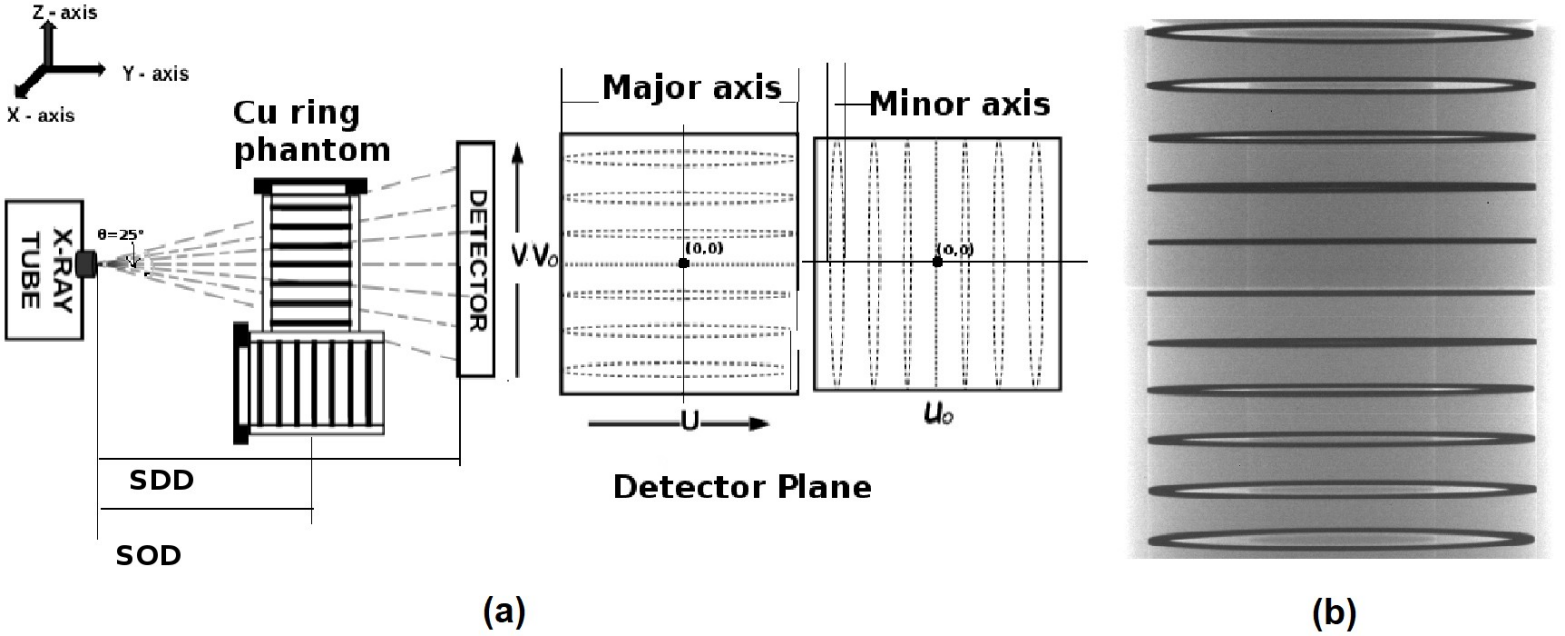
Approximate calibration method. (a) The copper ring phantom was kept parallel and orthogonal to the rotary axis in the micro-CT scanner. The detector’s center point(U_0_, V_0_) was estimated approximately from the radiographic images. Here, SOD refers to the distance between source to object, and SDD refers to the distance between source to the detector, (b) 2D projection image of the customized copper ring phantom scanned at the energy 35 kVp, 750 *μ*A, 2 fps is shown. The particular projection image shown was used to estimate V_0_.

The geometry of the micro-CT system was defined by the following. A)The location of the X-ray focal spot (which emanates a cone of X-rays) on the detector plane. B)The distance between the source i.e., focal spot and detector plane. C) The distance from the axis of the rotation of the rotary stage to the detector plane. D) The tilt of the detector plane. Accurate estimation of the aforementioned quantities was essential for accurate image reconstruction and was referred to as geometric calibration. We have used a well-established technique, as described by Kai Y et al., for our calibration [33]. However, before calibration, to reduce error, we position our rotary stage and detector as close as possible to the ideal setup. The ideal setup would be when the X-ray focal spot projects precisely to the center of the detector. The axis of rotation was parallel to the sides of the detector, and the rotation axis itself divides the detector into two halves when projected onto the detector plane. We use the copper ring phantom to get as close as possible to this ideal setup. The copper ring phantom consists of a series of copper rings of 50 *μ*m thickness stacked between acrylic disks inside a 30 mm diameter acrylic tube. As shown in Fig 7, projection images of the copper rings that were not close to the plane containing the focal spot and orthogonal to the detector plane appear like ellipses, while the copper rings that were on the plane or were close to the plane will be projected as straight lines. We affix the copper ring phantom so that the long axis of the phantom was parallel to the axis of rotation (indicated by the major axis in Fig 7) of the rotary stage. We then adjust the position of the detector till we get the straight-line projections approximately near the center point of the detector. We then flip the phantom ninety degrees (indicated by the minor axis in Fig 7(a)) and repeat the procedure. Note that this method was qualitative, and only measurements were made to check that the straight-line projections fall near the center coordinates of the detector.

**Fig 8:**
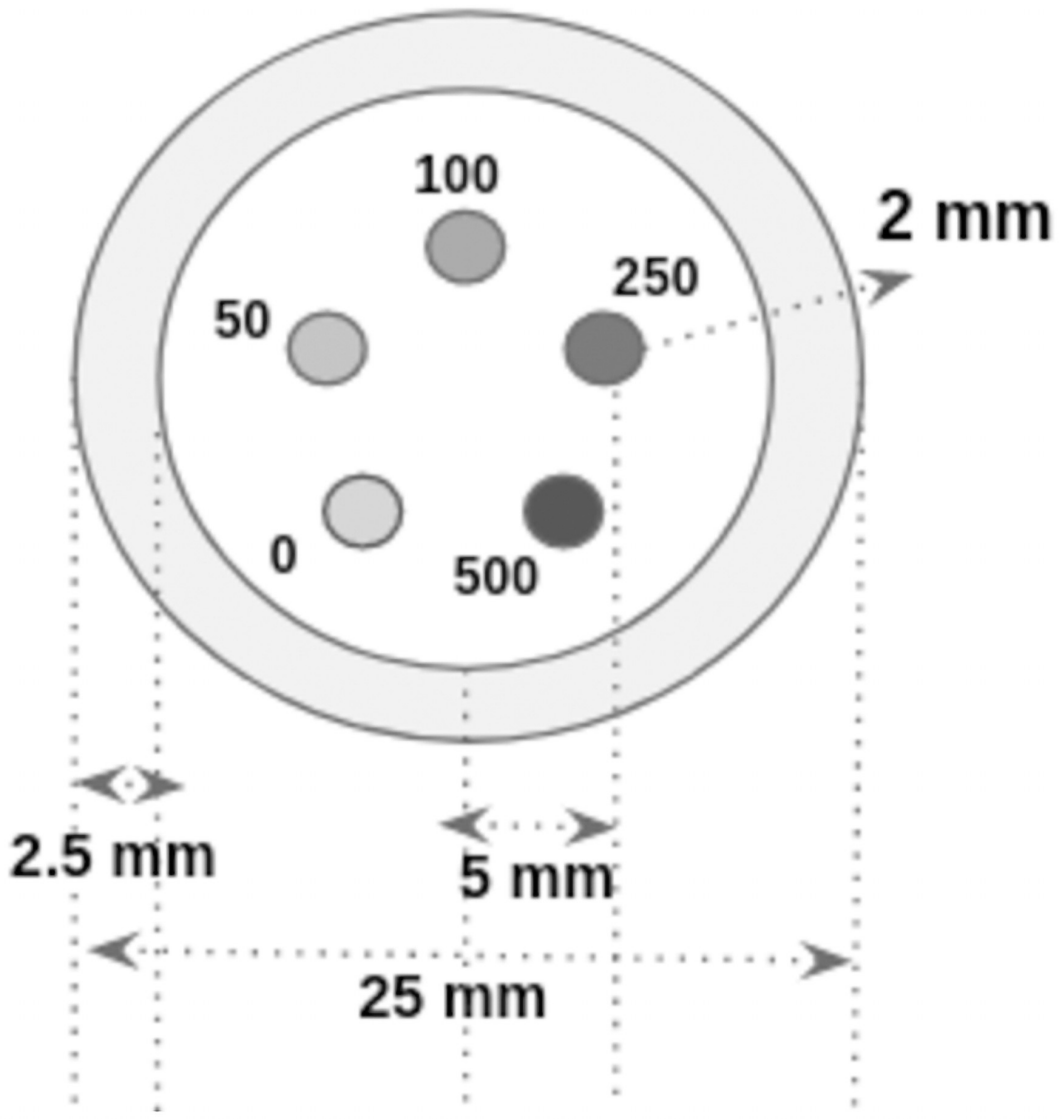
Micro-CT contrast phantom with HA rods. Cylindrical micro-CT contrast phantom had different concentrations of HA rods such as 0 mg/cc, 50 mg/cc, 100 mg/cc, 250 mg/cc, and 500 mg/cc. The micro-CT rods of diameter 2 mm and height 20 mm were placed circularly in the 40 mm height, 25 mm diameter solid acrylic customized phantom.

The customized micro-CT contrast phantom shown in Fig 8 was used to perform the system comparison study. The micro-CT contrast phantom has contrast rods of diameter 2 mm, length 20 mm (CIRS, tissue simulation, and phantom technology, USA). Various densities of hydroxyapatite (HA) contrast rods(0 mg/cc, 50 mg/cc, 100 mg/cc, 250 mg/cc, and 500 mg/cc) were placed circularly in the solid acrylic material(25 mm diameter). In our experiment, we took the projection images of a micro-CT contrast phantom (Fig 8). We reconstructed the cross-section, and compared it against the cross-sections reconstructed using a commercial scanner.

## Results

### Characterisation of X-ray imaging detector

#### Cascaded linear model

**Fig 9:**
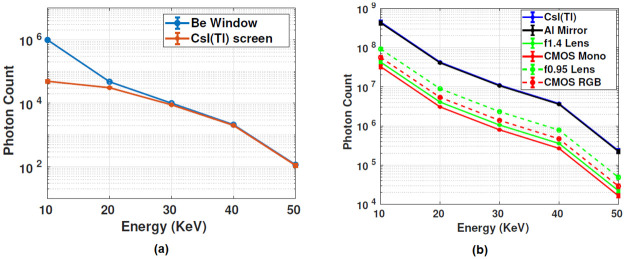
X-ray photons to light conversion. (a) Blue curve represents the number of X-ray photons exiting the Be window(courtesy: Source-ray inc., USA), the brown curve in the same graph is the X-ray transmission response of the CsI(Tl) screen (courtesy: Hamamatsu), (b) shows the attenuation of the light photons through various stages of the detector(*stage 2 to 4*). Here, the blue graph indicates the light photons created by the CsI(Tl) screen, the black curve indicates the reflection efficiency of the mirror for the input light photons(slightly overlapped with the blue curve), the green graph is the photon response of the relay lens(f1.4, f0.95), and the red curve indicates the light collection response of the CMOS sensor(Monochrome and RGB).

The scintillating screen in the *stage 1* had the quantum efficiency (*η*) of 0.825, and multiplying the quantum efficiency with the initial photons(q_0_) gives the average number of incident photons on the screen(Eq 1). The efficiency spectrum of collected photons at each stage in the cascaded system was plotted in Fig 9. Here Fig 9(a) is the plot between the total number of X-ray photons incident on the scintillator and its transmission efficiency for different X-ray energies. The transmission spectrum of the screen plotted for various X-ray energy was estimated from the manufacturer’s datasheet(courtesy Hamamatsu). The total number of visible photons obtained from the scintillating screen and the attenuation of the photons by the optical setup were shown in Fig 9(b).

#### Point spread function estimation

**Fig 10:**
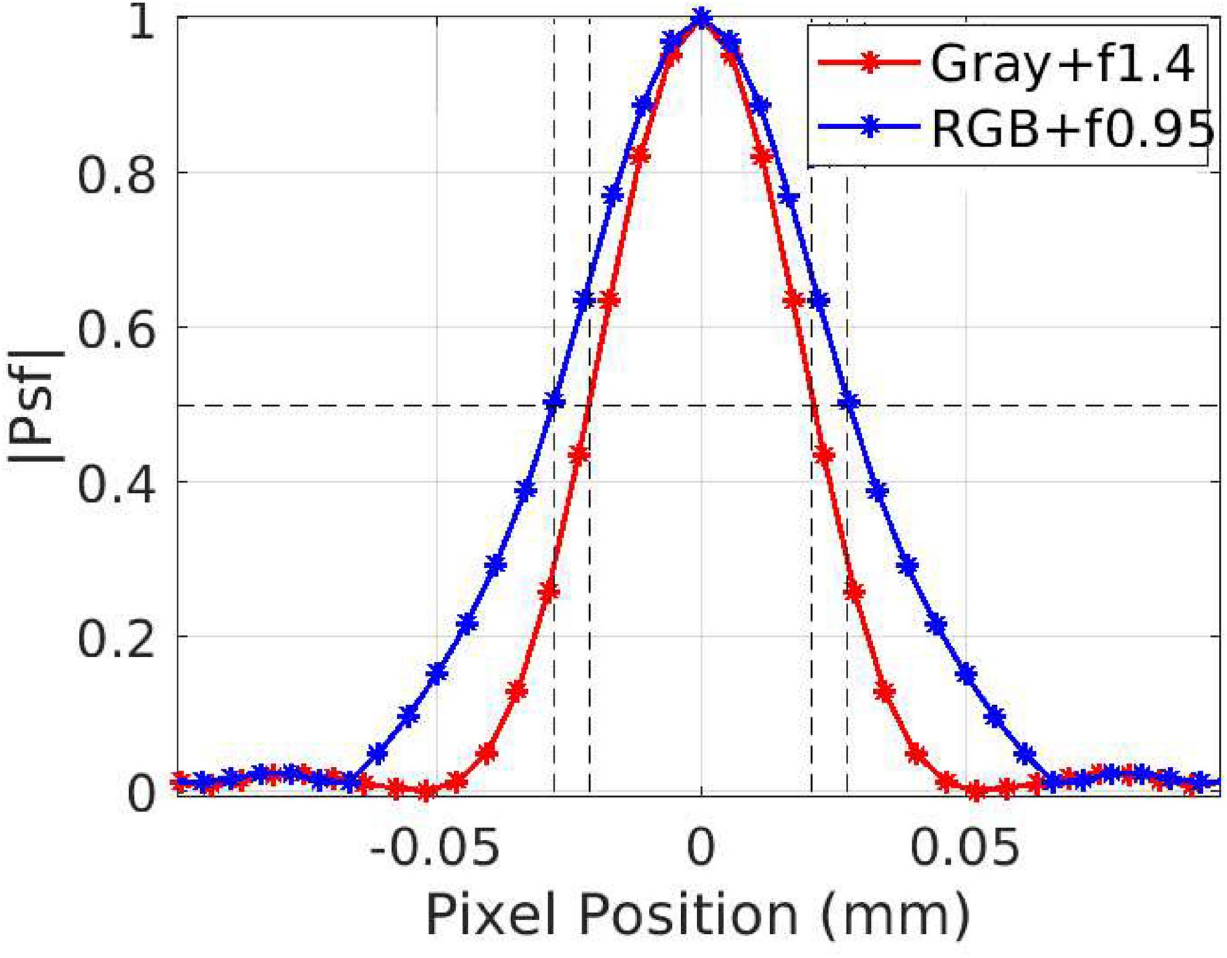
PSF of the OCX detector. The point spread function of the camera was observed using a 50 *μ*m diameter metal ball. Here, the RGB camera’s full width at half maximum(FWHM) was estimated as 53 *μ*m, and the monochrome camera was estimated as 44 *μ*m.

Fig 10 shows the PSF of the monochrome camera with an f1.4 lens and the RGB camera with an f0.95 lens. The RGB had a slightly wider PSF than the monochrome. Typically the RGB camera had lower quantum efficiency(QE), resulting in poor light collection efficiency. Especially in our case at 530 nm(green), the RGB camera had a QE of 59.7%, whereas the gray-scale had 82%. Even though the QE was low, the RGB camera had high frame rates and was also lower cost. The Bayer filter in the RGB camera also reduces the system’s light collection efficiency, and the demosaicing algorithm introduces errors during image formation. The full width at half a maximum of RGB was approximately 0.053 mm, and the monochrome was 0.044 mm. The detector’s spatial resolution characterized by the point spread function (PSF) also incorporates the finite focal spot size effect.

#### Detective quantum efficiency of the detector

**Fig 11:**
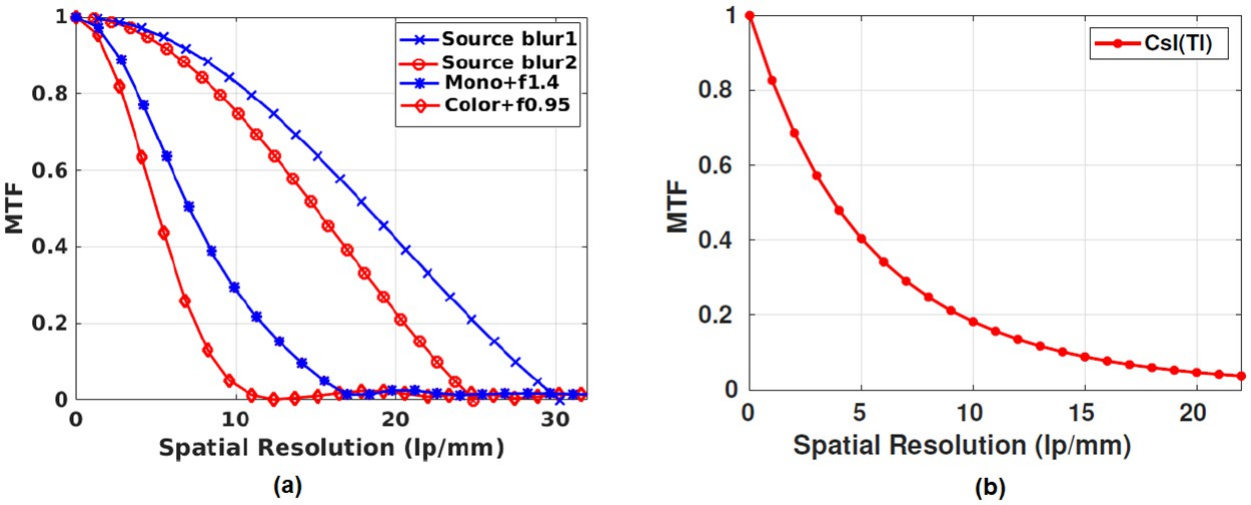
MTF plot for source and X-ray detectors. (a) MTF of the source blur plotted using the Eq 5. Here, source blur1 and source blur2 represent the geometrical unsharpness of the monochrome and RGB detector. Also, MTF of the monochrome and RGB based OCX detector is plotted in the same graph, (b) MTF curve of CsI(Tl) screen estimated using the screen specification as described by Ganguly A et al. [18].

**Fig 12:**
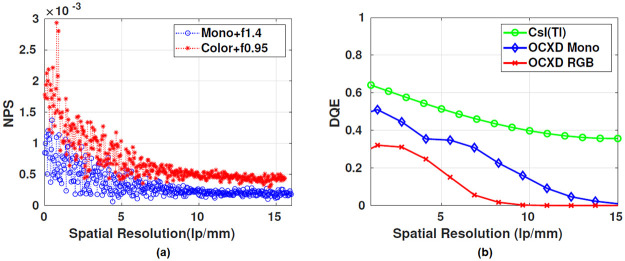
NPS and DQE spectrum of the detector. (a) Noise power spectrum obtained from the gain image, (b) DQE of the scintillator screen and the imaging system. Here, the DQE of the scintillator is plotted from the contrast data shared by the manufacturer(Fig 11), and the DQE of the imaging detector is estimated from the experimental data using metal ball PSF.

The MTF of the modeled source blur for different magnifications corresponding to the actual imaging setup of the RGB and the grayscale camera are shown in Fig 11(a) [27]. The blue curve corresponds to a magnification of 1.2, while the red curve corresponds to a magnification of 1.34 (Table 3). Also, the detector’s MTF is plotted in the same Fig 11(a) as blue and red plots corresponding to grayscale and RGB camera. The aforementioned figure shows that the grayscale had better MTF, 26% higher at 10 lp/mm. Using the datasheet shared by Hamamatsu, the MTF plot for CsI(Tl) was estimated and shown in Fig 11(b) *(Stage 2 under the subsection Cascaded linear model of the OCX detector)*. The noise power spectrum of the OCX detector in Fig 12(a) was obtained from the gain image. The gain image was captured with no object in the field of view, and the X-ray source was in *ON* condition. Substituting the observed NPS and detector’s MTF response in Eq 2, the DQE of the imaging system could be calculated. In Fig 12(b), the DQE corresponding to the RGB and monochrome camera are shown. Again, it was evident that the grayscale camera provides better DQE at a relevant spatial resolution range (i.e., 0 to 50 microns).

**Table 3:** Metal ball phantom image acquisition parameters.

	SOD(mm)	SDD(mm)	Magnification(*m*)
Monochrome	342.499	409.356	1.19
RGB	342.632	458.7	1.34

The geometrical values used in Table 3 were the system setting used to acquire the projection image of the metal ball phantom.

#### Spatial resolution estimated from the reference bar pattern

**Fig 13:**
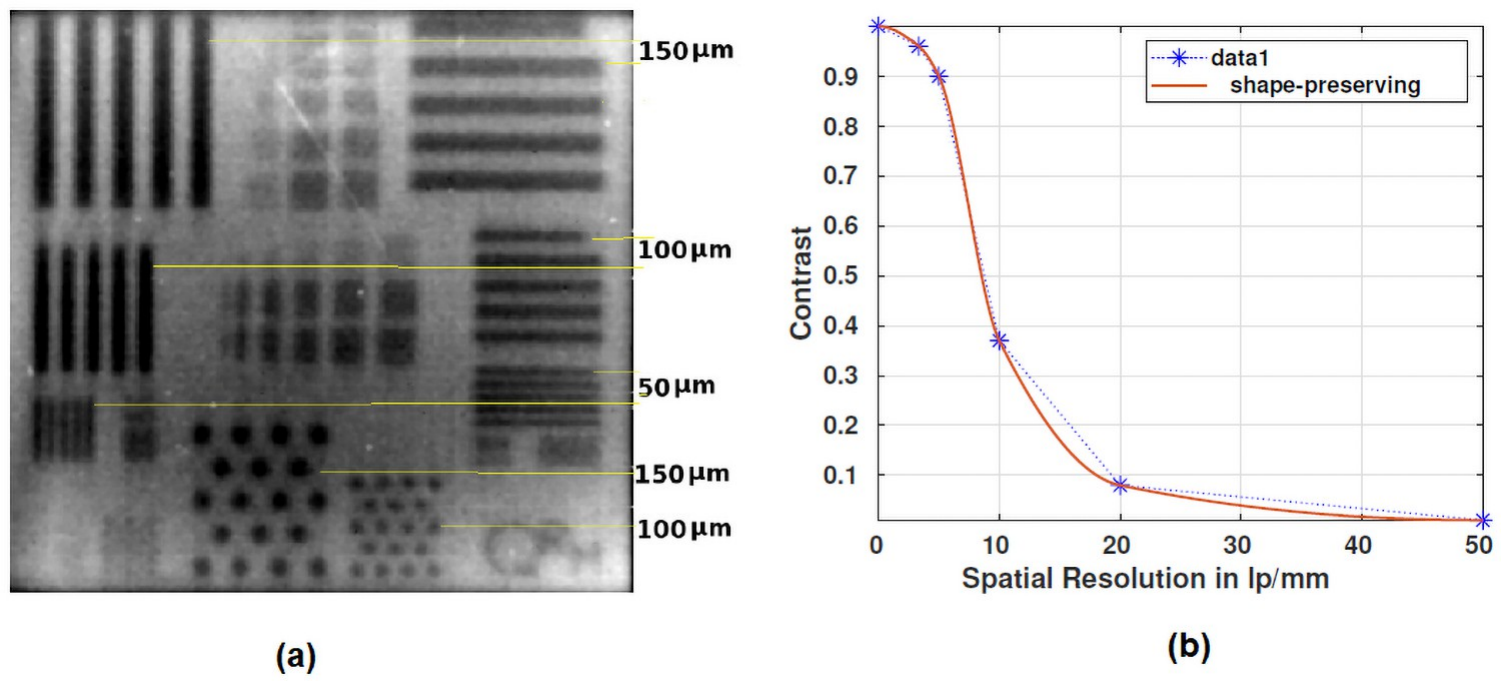
Spatial resolution of the OCX detector. (a) Projection image of the *QRM* bar pattern acquired at the maximum magnification of 8.3 and 35 kVp, 750 *μ*A, 5 fps as system setting, (b) MTF plot for the bar pattern. The contrast value in the MTF graph was obtained from the intensity profiles of line patterns at each spatial frequency in the projection image.

The projection image in Fig 13(a) shows the average of 100 frames acquired at a system setting of 35 kVp, 750 *μ*A, and 5 fps. The spatial bar pattern phantom contains 3.3 to 100 lp/mm line patterns in the silicon chip and at least 5 lines in each line pattern. MTF of the system was estimated from the line patterns in the projection image (Fig 13(a)). The difference in contrast of the pattern to the background region was given in the Eq 7.

$$\begin{array}{c} contrast=\frac{I_{max}-I_{min}}{I_{max}+I_{min}} \end{array}$$ (7)

where, *I*_*max*_ was the maximum intensity and *I*_*min*_ was the minimum intensity.

The above Eq 7 gives the contrast values of the line patterns obtained from the projection image, and thus the contrast at each spatial frequency(3.3 to 20 lp/mm) helps to plot the MTF of the system (Fig 13(b)). At 10 lp/mm, the contrast was recorded above 35%, and it started to decay fastly from 5 lp/mm.

### Fast frame rate studies

#### Estimating CNR from the phantom studies

**Fig 14:**
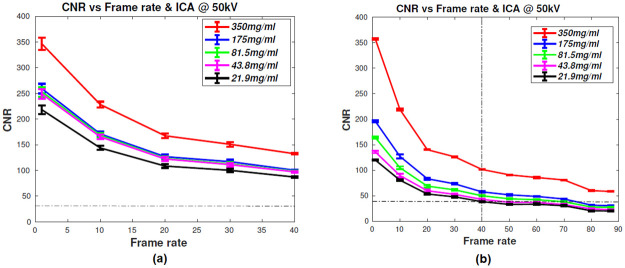
CNR plot for the fast frame rate studies. CNR is plotted as a function of frame rate for (a) monochrome and (b) RGB camera. The CNR study is performed with an iodinated contrast agent (ICA) at different dilutions and 50 kVp tube voltage. CNR value observed from the in-vivo studies is shown as dotted lines in the graphs.

*Iodine test phantom*. Fig 14 shows the variation of CNR as a function of frame rate. Here, the CNR was estimated using iodine test phantoms filled with the iodine contrast agents at different concentrations, and it’s explained in the *section Iodine test phantom to estimate the CNR of the detector*. As expected, the CNR decays as the number of frames increases. It was observed that for both grayscale and RGB cameras, the visibility of the phantoms was drastically reduced beyond 40 frames per second. The CNR estimated at high frame rates using the contrast-filled capillary tubes more than meets the Roses criterion [34]. The experiments were performed with iodine contrast concentrations which correspond to physiologically significant contrast concentrations.

**Fig 15:**
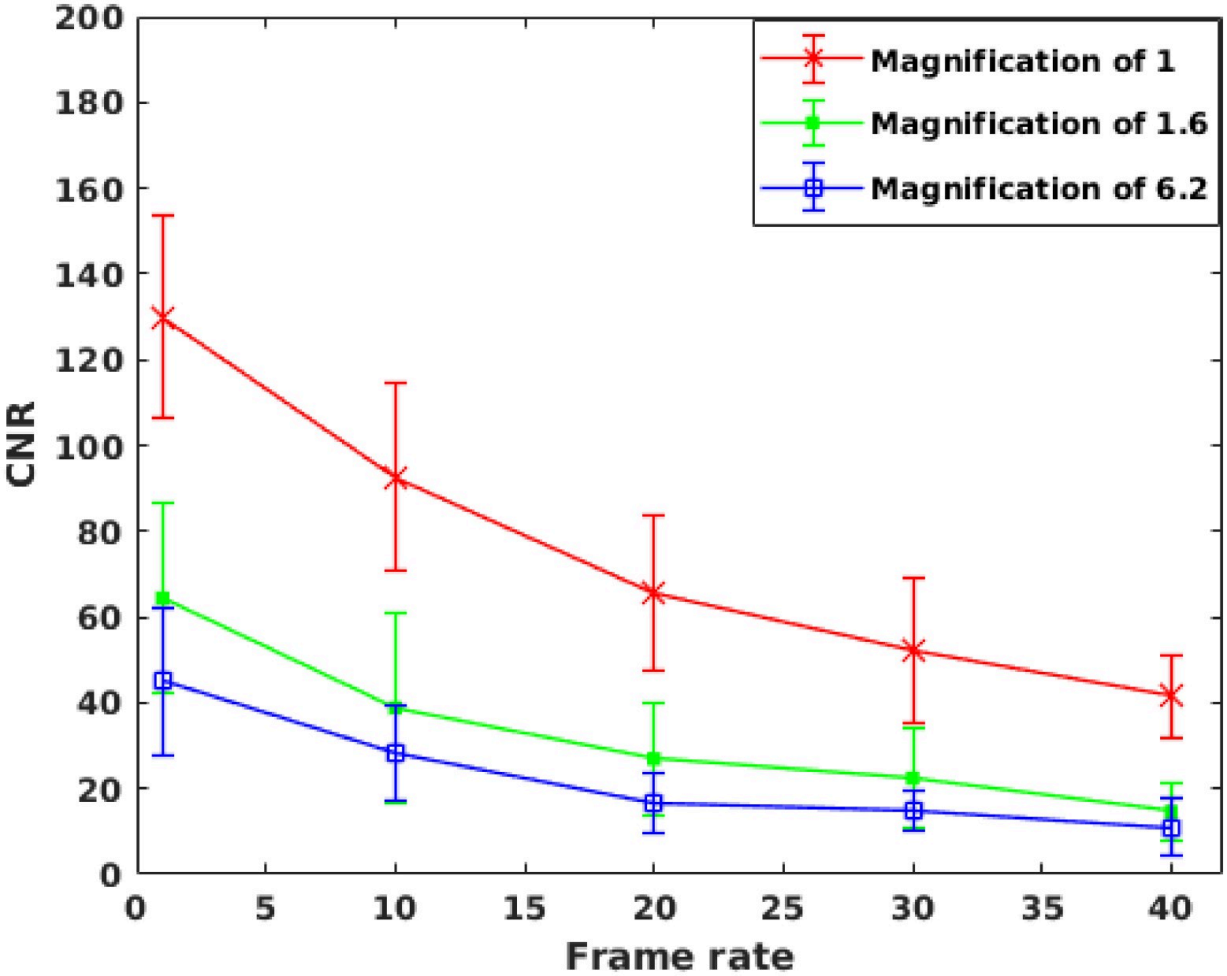
CNR graph of tungsten wire estimated from images acquired using the monochrome sensor. CNR graph of tungsten wire phantom imaged using the monochrome sensor at different magnification and frame rates. X-ray tube settings: 50 kVp, 900 *μ*A.

*Tungsten wire phantom*. Tungsten(W) wire phantom was used to study the high contrast resolution at various frame rates with the monochrome camera. The tungsten wire of 125-micron thickness was kept along the axis of a 30 mm diameter cylindrical acrylic phantom. The thickness of the wire corresponds to that of a typical size of the cardiac artery in mice. Fig 15 shows the CNR of W wire phantom at different magnification and imaged at 50 kVp, 900 *μ*A. Based on the experimental observation, the attenuation coefficient corresponds to 350 mg-I/ml at 50 kVp estimated as 0.8 cm^−1^ [35]. The tungsten’s attenuation coefficient at 50 kVp was 114.5 cm^−1^ (courtesy: NIST standard reference database). The high attenuation values for the W wire helps to estimate the high contrast resolution at high frame rates.

#### In-vivo cardiovascular imaging

**Fig 16:**
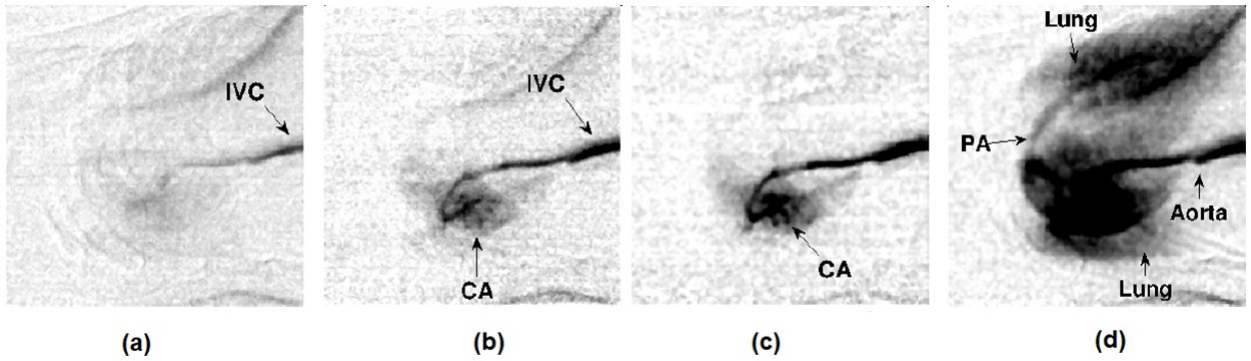
Experiment-I angiographic studies using monochrome camera. System setting: 50 kVp, 1 mA, 41 fps (a) arrival of ICA to the heart through IVC, (b) ICA gets in the right atrium, (c) the coronary arteries covered by the heart were visible, and (d) the pulmonary artery (PA) pumped out the ICA to lungs.

Fig 16(a), shows the arrival of ICA to the heart through inferior vena cava (IVC) at T1 = 0s, (b) ICA gets in the right atrium (RA), so the vena cava gets highlighted at T2 = 195ms, (c) the coronary arteries (CA) covered by the heart at T3 = 245ms, and (d) after some time, pulmonary artery (PA) pumps out the iodine to the lungs at T4 = 2s. Here T1 corresponds to the approximate time of the initial exposure, and T2, T3, and T4 are the selected frames to visualize the ICA’s post-arrival (S1 Video).

**Fig 17:**
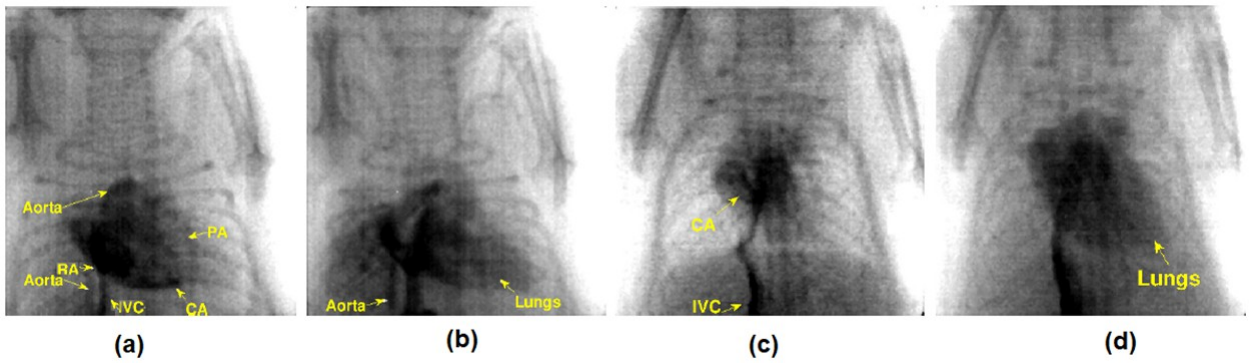
Experiment-II & III cardiac studies (mice-2 & 3) using RGB camera. System setting: 50 kVp, 1 mA, 40 fps. Experiment with mice 2 is shown in (a) and (b), iodine highlighting the IVC, RA, CA, PA, aorta, and lungs. A similar experiment with mice 3, is shown in (c) and (d), where the coronary arteries and lungs are highlighted.

Fig 17(a) shows the iodine entering into the right atrium (RA) through the IVC, and then it enhances the contrast of right coronary arteries (RCA) at T1 = 2s, (b) at T2 = 4.73s, ICA highlights the heart’s apex and pulmonary artery (PA), which supplies deoxygenated blood to the lungs and pumps oxygenated blood from the aorta, (c) at T1 = 3.1s, the ICA highlights the IVC, and (d) at T2 = 8.9s, the contrast enhances the right atrium, aorta, and lungs.

The fluoroscopic images were obtained by exposing the rodent for a short time interval (at least 2 minutes). The selected images were denoised using a median filter and contrast adjusted (in the range of 45 to 140) in MATLAB. Post-processed fluoroscopy images are shown in Figs 16 and 17. The diaphragm movement method helps to calculate the heart and breath rates of rodent I as 381±29 beats per minute and 71±6 breaths per minute. The in-house small animal ECG monitoring circuit estimates rodents II and III’s heart and respiratory rate as 418±32 beats per minute, 365±11 beats per minute, and 116±7 breaths per minute, 46±6 breaths per minute.

*Dose*. RTI (RT 100 dose probe, Sweden) dosimeter was used to measure the dose rate of the animals. Animals are exposed to the dose of 93.3 *μ*Gy per second and the cumulative dose for two minutes angiographic study was 11.2 mGy.

Around 200 *μ*l of ICA (350 mg-I/ml) was injected into the rodent for the angiographic studies. The average mouse has approximately 2 ml of blood volume, which translates to 35 mg-I/ml in-vivo, assuming that the iodine contrast agent was diluted uniformly. The CNR values from the in-vivo studies were 32.84±5.23 and 40.16±4.88 (mentioned as dotted-dash lines in Fig 14(a) and 14(b)). The CNR values were calculated by considering a region (5x5 pixel) of interest in the inferior vena cava (IVC) with iodine contrast agent (signal) and soft tissue without contrast agent (background)(Eq 6). From the Figs 16 and 17, the diameter of IVC was estimated as 0.7 mm and the aorta in the range of 0.4 to 0.5 mm. It was estimated using the number of pixels occupied by the ICA in the processed images. Beyond 40 fps, the capillary tubes were barely visible, and CNR could not be estimated reliably with such images. However, in-vivo mouse cardiac angiography studies showed high CNR even at a high frame rates due to higher concentrations of iodinated contrast agents in the venous blood. The grayscale camera used to perform these experiments could not be operated at frame rate higher than 40fps. The RGB camera provided poor image quality at higher frame rates due to the low QE of the Bayer array. We observe the significant impact of the reduction in photon fluence as the frame rates approach 40fps and higher in both these sensors.

### Micro-CT contrast HA rod phantom

**Fig 18:**
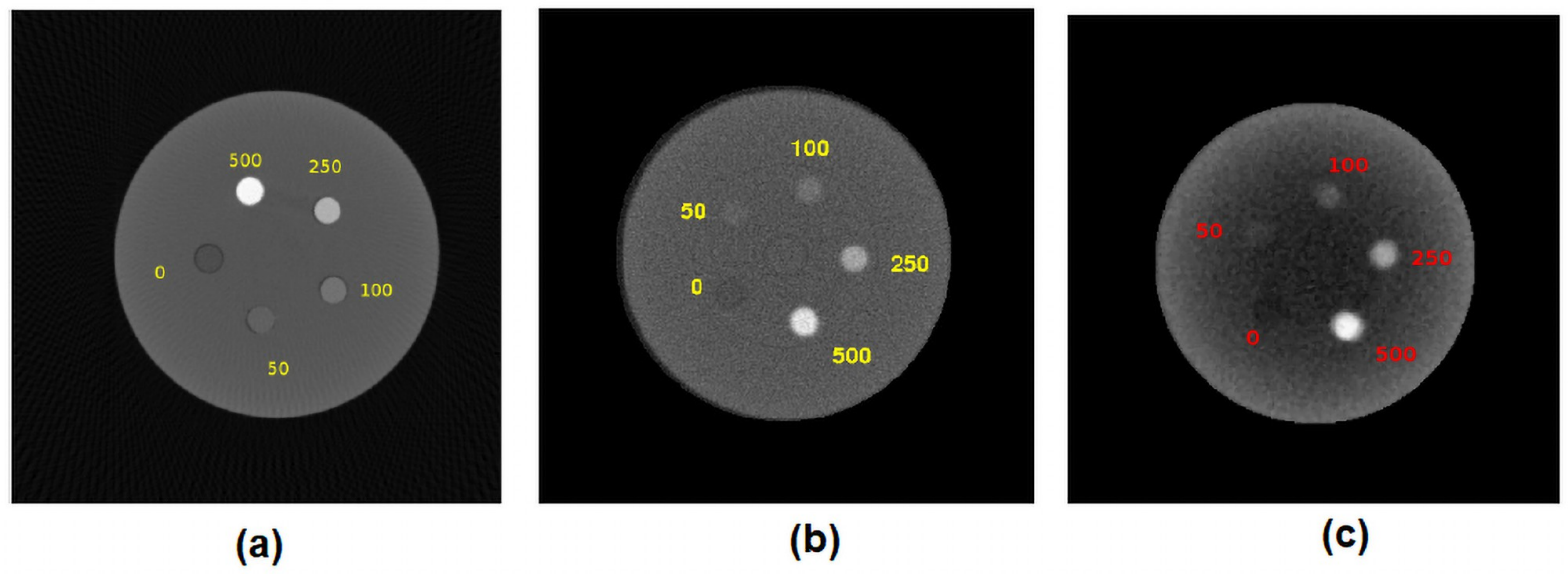
Comparison against the commercial scanner. Cylindrical contrast phantom with different concentrations of HA micro-CT rods scanned using our micro-CT and GE scanner. (a) reconstructed image from the GE scanner, (b) image reconstructed from the micro-CT scanner using monochrome CMOS sensor, and (c) image reconstructed from the scanner using RGB CMOS sensor. The phantoms were reconstructed using an in-house FDK reconstruction algorithm in python.

The OCX detector’s performance was compared with a *GE phoenix industrial high-resolution CT & X-ray system*. Contrast phantom was customized with the micro-CT contrast rods with different concentrations of hydroxyapatite (HA) such as 0 mg/cc, 50 mg/cc, 100 mg/cc, 250 mg/cc, and 500 mg/cc placed in a customized phantom. For Fig 18(a) GE scanner input parameters were calculated with the help of calibration phantom as 278.706 mm (SOD), 797.145 mm (SDD), (U_0_, V_0_) as (98.595, 101.069) mm and the in-plane rotation of detector (*η*) was 0.0215°. Projection image of the contrast phantom from in-house micro-CT was acquired at 50 kVp, 1000 *μ*A, 500 ms exposure time. Images shown in the Fig 18(b) and 18(c) were reconstructed using 180 projection images and a magnification factor of 2.86. The center point of the detector (U_0_, V_0_) was (23.87, 23.9) mm. Both the phantoms were scanned with the same energy, current, and frame rate. The phantoms were reconstructed at 100 *μ*m voxel size in a 36x36x36 mm volume using the standard feld kamp davis (FDK) algorithm implemented in python [36].

## Discussion and conclusion

We characterized our system using conventional metrics like DQE, NPS, MTF, and CNR experiments pertinent to the in-vivo studies. The DQE of the system(both RGB and grayscale) was calculated using the estimates of the MTF and NPS. It was clear that the optical coupling(g_*oc*_) degrades the DQE of the detector. At resolutions higher than 100 *μ*m, the DQE of the monochrome OCX detector decreases gradually, and for RGB OCX detector decreases rapidly (Fig 12). This could be due to multiple channels (RGB) and interpolation carried out in a Bayer array. However, even for the grayscale camera, the DQE was considerably lower for frequency values greater than 10 lp/mm i.e., the higher frequencies were degraded more, especially those of interest in in-vivo pre-clinical angiography studies. For instance, we expect the coronary arteries to be 100 microns or less, and in that regime, the DQE of the grayscale camera-based system is still not good enough for clear visualization of mouse coronary arteries.

A combination of high frame rates, lower X-ray tube peak voltage, and tube current leads to degradation of DQE in the resolution range of interest. In addition, the use of RGB CMOS sensors also seems to degrade performance. This can be observed in our CNR phantom studies and in the in-vivo angiographic studies, where there was evident degradation in the image quality (qualitative) compared to the system using the grayscale camera. Experiments can be performed with higher frame rates if the X-ray tube can be operated at higher peak voltage and with higher X-ray output flux. Blurring due to motion can be further reduced by using a combination of ECG and respiratory gating, leading to better image quality in the resolution range of 50 microns and higher.

The in-vivo studies show significant differences in image quality between the grayscale and RGB sensor-based X-ray cameras. A comparison of various frames acquired after injection of contrast indicates better contrast in the grayscale CMOS sensor-based cameras. However, neither of the cameras could delineate coronary arteries smaller than 100 microns in diameters. As seen in the images Figs 16 and 17, only the large arteries like the aorta are visible with better overall contrast when images were acquired using the grayscale CMOS sensor-based camera. The CNR studies using the test phantoms served as a pointer to the in-vivo studies. The high frame rates were unavoidable since the mouse heart rates of several hundred beats per second and our estimates based on ECG confirm the same (7 bps). A back-of-the-envelope calculation would indicate that at 7 bps, one needs to acquire images at 40 fps to capture a single heartbeat cycle. The use of respiratory and ECG gating, as stated earlier, could help improve the contrast by freezing the motion.

For 3D imaging, it was clear that the grayscale sensor-based camera was suitable for tomographic acquisition. The reconstructed phantom images were compared favorably (qualitative) against a high-end commercial system. However, we should point out that the problems that occur with high frame rates and lower energies can be compensated to some extent by average over multiple frames for 3D imaging. In addition, for specimen imaging, motion artifacts were absent, leading to better images. The RGB CMOS sensor-based camera for 3D micro-CT imaging and reconstruction shows severe beam hardening artifacts caused by low energy X-ray photon attenuation through the object and low SNR at the RGB CMOS sensor.

We used cameras capable of imaging at high frame rates (>30 fps suitable for sampling a single cardiac cycle), fit our cost constraint, and have high quantum efficiency in the wavelength of interest. There were other constraints like sensor size and pixel size, which would determine the final resolution of the system. These were also considered but were secondary. In summary, the system can be characterized with the following metrics. The optical setup of the OCX detector had a light coupling efficiency of 0.0145, and the detector designed with a monochrome camera had 35–40% contrast at the spatial frequency of 10 lp/mm (Fig 13). The detector collects 17% of light photons from the cascaded stages at 10 lp/mm (Fig 12). The proposed detector could observe the minimum dilution of 21.9 mg-I/ml at 50 kVp (Fig 14) and also delineate arteries of size >0.4 mm in diameter.

Several groups have explored the use of high CCD camera-based optically coupled X-ray detectors for fast frame rate imaging along with clinical X-ray sources or synchrotron sources. These methods tend to increase the financial, safety, and infrastructural costs of performing in-vivo experiments. Using off-the-shelf components to build a multi-purpose system, as we have described, poses a unique set of challenges that have been highlighted with various experiments. Primarily the trade-off between costs and image quality and, secondly, the trade-off between temporal resolution and spatial resolution when performing angiography studies. In the S1 Appendix, we have compared the system behavior with the cost and performance of the system. The comparison shows that the proposed system had a good compromise for routine imaging(Table 5 in S1 Appendix). We conclude that adjusting the aforementioned metrics appropriately can lead to better imaging performance in terms of CNR and DQE. Future work would focus on improving the optical coupling and higher energy microfocus X-ray tubes for an affordable multi-purpose X-ray imaging and micro-CT system.

## Supporting information

S1 Appendix (PDF)

S1 Video Cardiac angiography study. The supplementary video contains a continuous sequence of images acquired during ICA injection into the rodent-I. In the video, there are three images, namely original image, mean image, and RPCA processed image. The original image was the sequence of unprocessed projection images with the iodine contrast agent(acquired during the angiographic study). The mean image was the sequence of radiographic images without the contrast agent(acquired before the angiographic study). To get the RPCA processed image, we followed two steps 1.The original image was subtracted from the mean image and 2.The resultant image was enhanced using the inbuilt robust principal component analysis (RPCA) algorithm in MATLAB.
(MP4)
