# Supplementary material for: X-ray scintillator lens-coupled with CMOS camera for pre-clinical cardiac vascular imaging—A feasibility study
Source: PLoS One. 2022 Feb 11;17(2):e0262913. doi: 10.1371/journal.pone.0262913 (PMC8836319; doi:10.1371/journal.pone.0262913)
Supplement: S1 Appendix — (PDF) [file pone.0262913.s001.pdf]

Appendix

Table 4. Abbreviations for the acronyms frequently used in the manuscript

**Acronyms**	**Abbreviations**
CCD	Charge-coupled device
CMOS	Complementary metal oxide semiconductor
CNR	Contrast to noise ratio
CTF	Contrast transfer function
DQE	Detective quantum efficiency
FPD	Flat panel detectors
ICA	Iodinated contrast agents
MTF	Modulation transfer function
NPS	Noise power spectrum
OCX	Optically coupled X-ray detector
PC	Personal computer
PMMA	Poly (methyl methacrylate)
PSF	Point spread function
RPCA	Robust principal component analysis

Table 5. Performance of the system tabulated with approximate cost

**Reference**	**Typical components used**	**Typical capabilities**	**Estimated minimum cost of the detector**
Helen XF [1]	Phosphor screen, lens, CMOS camera	best spatial resolution $100\mu m$	Economical ($5000 - 7000)
Yang M et al., Ming DL et al., [2], [3]	Fiber coupled scintillator, lens, CCD camera	resolution will be in the range of 50 - 100 μm	Moderate ($7000 - 12000)
Jain A et al., [4]	Scintillator, fiber coupler, image intensifiers, CCD camera	very high resolution of 10 - 50 μm	Expensive (> $12000)

Helen XF, developed an x-ray detector with a phosphor screen of size 24x36 mm, f1.4 lens and CMOS camera. The system showed an MTF of 50% at 2.5 lp/mm operated at 30 fps. The X-ray tube energy was 130 kVp, 0.5 mA during the imaging [1].

Yang M et al., designed the x-ray detector for CT small animal imaging with a phosphor screen, optical mirror, two sets of lenses, an image intensifier, and an expensive CCD camera. The small animal imaging was performed at X-ray energy 50 kVp, the exposure level of 6 $mAs/frames$, and the resultant image acquired at the spatial resolution of 32 μm [2].

Ming D L et al., performed small animal angiographic imaging using gadox screen coupled with the expensive CCD camera, and the system had high SNR at 70 kVp x-ray energy. The angiographic studies were done at 30fps with the spatial resolution of $< 100\mu$m, and also a high concentration of iodine contrast agent (370 mg-I/ml) was injected into the rat [3].

Jain A et al., designed an image intensifier-based x-ray detector using CsI(Tl) screen, fiber coupler, and expensive CCD camera. The developed detector had 10% quantum efficiency at 5 lp/mm and imaged the stent of rabbit (average size imaged the stent of rabbit (average size 180 micron [5]) [4].

References

1. Helen XF. Lens-coupled X-ray imaging systems. Ph.D. dissertation, The University of Arizona. 2015 May.

2. Yang M, Chris CS, Xinming L, Mustafa CA, Tianpeng W, Lingyun C, et al. Comparison of two detector systems for cone beam CT small animal imaging - a preliminary study. Medical Imaging. 2006 Mar;6142:1–10. https://doi.org/10.1117/12.656690

3. Xiaolan W, Dirk M, Katsuyuki T, Douglas JW, Bradley EP and Eric CF. Material separation in X-ray CT with energy resolved photon-counting detectors. Medical physics. 2011 Feb;38(3):1534–1546. https://doi.org/10.1118/1.3553401

4. Jain A, Bednarek DR, Ionita C, Rudin S. A theoretical and experimental evaluation of the microangiographic fluoroscope: A high-resolution region-of-interest X-ray imager. Medical physics. 2011 June;38(7):4112–4126. https://doi.org/10.1118/1.3599751

5. Ruoxi Z, Shuyuan C, Hui Z, Qi L, Jianpang X, Qi Z, et al. Effects of Methotrexate in a Rabbit Model of In-Stent Neoatherosclerosis: An Optical Coherence Tomography Study. Scientific Reports. 2016 Sept;6(33657). https://doi.org/10.1038/srep33657
